# Editorial: Peripheral stimulation: neuromodulation of the central nervous system through existing pathways

**DOI:** 10.3389/fnins.2023.1285474

**Published:** 2023-10-11

**Authors:** Musa Ozturk, Julio Cesar Hernandez-Pavon, Alexander Kent, Jose L. Pons, Ilknur Telkes, Arjun Tarakad

**Affiliations:** ^1^Iota Biosciences, Alameda, CA, United States; ^2^Department of Psychological Sciences, College of Arts and Sciences, Kansas State University, Manhattan, KS, United States; ^3^Cala Health, Burlingame, CA, United States; ^4^Shirley Ryan AbilityLab, Chicago, IL, United States; ^5^Charles E. Schmidt College of Medicine, Florida Atlantic University, Boca Raton, FL, United States; ^6^Department of Neurology, Baylor College of Medicine, Houston, TX, United States

**Keywords:** peripheral stimulation, neuromodulation, bioelectronic medicine, central nervous system, neurological disorders

Neural stimulation presents a promising avenue for treating neurological disorders, offering an alternative or complement to the traditional pharmaceutical approaches. By delivering targeted stimulation specifically to the brain region that is causing the pathology, the potential for a wide range of adverse effects from drug treatment may be reduced. However, some conventional brain stimulation methods, such as deep brain stimulation (DBS), involve high-cost, high-risk surgeries to implant electrodes into the brain and a battery to the chest, with a wire connecting them to deliver electrical stimulation.

In this Research Topic, we asked the question: can we utilize the existing “wires” -peripheral nerves- in the body for stimulating the brain instead? Specifically, the overlapping structures between peripheral pathways (e.g., sensorimotor and vasovagal pathways) and the central circuits of the brain (e.g., cerebellothalamic and reward circuits) may enable such utilization. The articles in this collection aim to illuminate some of the possibilities of peripheral stimulation as a non-invasive neuromodulation method for the central nervous system (CNS).

Advancements in various techniques, such as electrical, vibrotactile, and audio/visual stimulation, have piqued interest in their potential applications for treating neurological and autonomic disease indications. However, sometimes to go forward, one must look back. Li et al. discuss how acupuncture, a traditional form of peripheral nerve stimulation used for thousands of years, can regulate the autonomic nervous system (ANS) to treat disorders such as migraine, pain, and inflammation. This review article highlights that acupuncture activates sensory nerves and spinal pathways to modulate a complex network of autonomic nuclei within the brain, such as the hypothalamus, brainstem nuclei, and cortical regions. This, in turn, regulates sympathetic, parasympathetic, and enteric efferents to restore homeostasis and alleviate symptoms. Further study on the specific neural pathways and mechanisms involved for different acupoints, stimulation parameters, and conditions could help optimize the clinical outcomes. Overall, the modulation of ANS represents an important link between acupuncture and its therapeutic effects.

Acupuncture is not the only way peripheral stimulation can modulate the ANS though, as Garcia et al. use electrical stimulation to do so. This article investigates the effects of different respiratory-gated auricular vagus afferent nerve stimulation (RAVANS) frequencies on blood pressure and heart rate variability in hypertensive patients. The RAVANS system stimulates the auricular branch of the vagus nerve in the ear only during the exhalation phase of the breathing cycle. RAVANS shows frequency-dependent effects, with 100 Hz stimulation significantly lowering heart rate and blood pressure compared to sham, especially in some subgroups. This non-invasive approach likely acts on the medullary vagal complex to increase parasympathetic activity and/or reduce sympathetic drive. The findings demonstrate that peripheral stimulation can modulate central circuits to treat disorders like hypertension and that the stimulation parameters need to be optimized to achieve the greatest effects on the modulation of physiological and clinical outcomes.

Peripheral nerve stimulation is not always a replacement to direct brain stimulation but can also be complementary. Koseki et al. investigate the combined effects of neuromuscular electrical stimulation (NMES) and transcutaneous spinal direct current stimulation (tsDCS) on the neural plasticity of the CNS in healthy participants. NMES activates peripheral sensory nerves while tsDCS modulates spinal cord excitability, and their combination induces longer-lasting effects compared to either stimulus alone. Results showed an increased corticospinal excitability for at least 60 min, reduced intracortical inhibition, and decreased spinal reflex excitability immediately after stimulation. The findings suggest that the enhanced motor cortex plasticity by combined stimulation could help develop new neurorehabilitation techniques. However, further research is needed in patients with neurological conditions such as those with CNS lesions.

Aside from its potential for novel therapeutic applications, peripheral stimulation can be utilized for diagnostic purposes as well. Fuksa et al. use audio/visual stimuli with various complexities to examine functional changes in the brain related to age, hearing loss (presbycusis), and tinnitus. The authors utilized functional magnetic resonance imaging (fMRI) to investigate brain activity during different types of acoustic and visual stimulations, and their combinations. The results demonstrate that the aforementioned pathologies as well as the stimulus complexity influence lateralization and activation patterns in the limbic system and the auditory cortex. The authors conclude by recommending types of audiovisual stimulation better suited to identifying between presbycusis and tinnitus, alluding to the diagnostic use of peripheral stimulation.

Suk et al. take peripheral stimulation to another level by suggesting a whole-body stimulation paradigm. The authors have previously reported the benefits of non-invasive visual stimulation using 40 Hz light-flicker and were curious if their findings were modality dependent. In the current article, similar to the visual stimuli, they observe that non-invasive whole-body vibrotactile stimulation at 40 Hz improves motor performance, induces neural activity, and reduces pathology in the somatosensory and motor cortices of mouse models of neurodegeneration. The findings demonstrate that non-invasive sensory stimulation at 40 Hz, engaging multiple modalities like vision and touch, represents a promising therapeutic strategy for mitigating pathology and improving performance in neurodegenerative diseases. While the study focuses on animal models, it implies the potential for similar strategies in human patients, although additional research is needed to establish the clinical applicability and efficacy of the approach.

No matter what modality is used for peripheral stimulation, it will be more effective if the peripheral nerves are healthy. Wang et al. explore the use of a regenerative peripheral nerve interface (RPNI) as a neuroprotective tool for injured nerves. RPNI involves implanting the proximal nerve stump into a free muscle graft, promoting synaptogenesis and sensory receptor reinnervation. The article demonstrates that RPNI rescues sensory neuron loss and apoptosis in dorsal root ganglia (DRG) neurons after sciatic axotomy in adult rats. They conclude that RPNI helps prevent neuronal loss after nerve injury, likely by re-establishing retrograde transport of neurotrophins from the reinnervated muscle back to the DRG. This provides a potential therapy to improve outcomes of delayed peripheral nerve repair by protecting injured sensory neurons from retrograde degeneration.

To conclude, we believe there is a significant untapped potential in neuromodulation through peripheral nerves. As the compendium of work presented in our Research Topic demonstrates, such therapies can derive inspiration from both novel technologies, such as regenerative nerve interfaces, and ancient traditional practices, such as acupuncture. We are particularly pleased that the articles in this Research Topic cover different sensory modalities, such as electrical, somatosensory, and audiovisual. Especially with the developments in non-invasive approaches such as transcranial magnetic stimulation (TMS), electro- and magneto-encephalography (EEG/EMG), the underlying mechanisms and pathways behind various peripheral stimulation approaches can be explored without surgical experiments. We look forward to ongoing interest in developing peripheral stimulation further, both with existing and new modalities, and providing a less-invasive alternative for the drug-resistant patient populations suffering from neurological disorders.

## Author contributions

MO: Writing—original draft, Writing—review and editing. JH-P: Writing—review and editing. AK: Writing—review and editing. JP: Writing—review and editing. IT: Writing—review and editing. AT: Writing—review and editing.

